# Pre-existing canine anti-IgG antibodies: implications for immunotherapy, immunogenicity testing and immunoassay analysis

**DOI:** 10.1038/s41598-020-69618-3

**Published:** 2020-07-29

**Authors:** Daniel Bergman, Camilla Bäckström, Helene Hansson-Hamlin, Anders Larsson, Bodil Ström Holst

**Affiliations:** 1 Department of Clinical Sciences, Swedish University of Agricultural Sciences, 750 07 Uppsala, Sweden; 2 Department of Medical Sciences, Uppsala University, 751 85 Uppsala, Sweden

**Keywords:** Immunotherapy, Autoimmunity, Immunological techniques, Drug development

## Abstract

One of the most enigmatic features of humoral immunity is the prevalent presence of circulating autoantibodies against IgG. These autoantibodies consist of several subsets, including rheumatoid factors, anti-Fab/anti-F(ab′)_2_-autoantibodies, and anti-idiotypic antibodies. Anti-IgG autoantibodies can impair the safety and efficacy of therapeutic antibodies and interfere with immunogenicity tests in clinical trials. They can also cross-react with allospecific IgG, presenting as heterophilic antibodies that interfere with diagnostic immunoassays. Owing to these factors, recent years have seen a resurgent interest in anti-IgG autoantibodies, but their underlying clinical significance, as well as biological roles and origins, remain opaque. Increased knowledge about canine anti-IgG autoantibodies could facilitate the development of canine immunotherapies and help in understanding and counteracting immunoassay interference. This study investigated the clinical significance and interconnection of heterophilic antibodies, anti-Fab, and anti-F(ab′)_2_-autoantibodies in dogs. We performed a 2-year prospective follow-up of dogs with heterophilic antibodies and analyzed serum for anti-Fab and anti-F(ab′)_2_-autoantibodies. Canine heterophilic antibodies can persist for at least 2 years in serum. A widespread occurrence of anti-Fab and anti-F(ab′)_2_-autoantibodies was found, with reactivity to cryptic epitopes in the IgG hinge region and sporadic cross-reactivity with mouse IgG. Canine anti-Fab and anti-F(ab′)_2_-autoantibodies are thus potential sources of clinical immunogenicity and immunoassay interference.

## Introduction

Immunotherapy has revolutionized the treatment of previously incurable diseases. By the end of 2019, 79 therapeutic monoclonal antibodies (mAbs) had been approved by the US Food and Drug Administration (FDA) for the treatment of a variety of human diseases^1^^,^ but only a handful of veterinary mAbs are approved, including two for the treatment of cancer in dogs^2^. One of several factors holding back this development is a lack of basic information about the canine immune system, including factors influencing the immunogenicity of therapeutic antibodies. Immunogenicity is a significant barrier to the approval of therapeutic mAbs and is only detectable after years of basic research, development and preclinical studies. Thus, accurate prediction of immunogenicity is one of the keys to successful drug development. Because immunological tolerance is highly species-specific, mAbs developed for human patients are immunogenic in animals and not suitable for use in veterinary medicine. Therapeutic mAbs are therefore normally speciated (humanized, caninized or felinized) to reduce their immunogenicity, but despite these efforts, all therapeutic mAbs elicit immune responses in some patients^3^.

Pre-existing anti-IgG antibodies in treatment-naïve patients are another source of immunogenicity. This umbrella term includes antibodies against autologous IgG, such as rheumatoid factors that bind to the Fc region of IgG. There are also anti-Fab/anti-F(ab′)_2_-autoantibodies that bind to IgG fragments obtained by enzymatic cleavage in vitro: Fab-fragments if the IgG molecule is cleaved in the upper hinge region and F(ab′)_2_-fragments if it is cleaved in the lower hinge region. To date, anti-IgG antibodies have been poorly researched in dogs, and canine anti-Fab and anti-F(ab′)_2_-autoantibodies have previously not been described. These antibodies can interfere with therapeutic antibodies, particularly with antibody fragments^4^^,^ and with immunogenicity tests in clinical trials^5^. A switch on this relationship is that the presence of pre-existing anti-Fab and anti-F(ab′)_2_-autoantibodies is associated with invasive disease. According to a recent theory, anti-F(ab′)_2_-autoantibodies can form in response to in vivo cleavage of IgG by proteolytic enzymes associated with cancer and autoimmune diseases^6,7^. We have shown that Bernese mountain dogs, a breed well-known for its high prevalence of cancer, also show a high frequency of antibodies targeting F(ab′)_2_-fragments of mouse IgG^8^. The Fab regions of canine and murine IgG contain constant heavy and light chains with approximately 60–70% interspecies sequential homology^9^^,^ so these antibodies may be cross-reactive autoantibodies that present as heterophilic antibodies when they bind to mouse IgG. Although canine heterophilic antibodies have received increased attention in recent years^8,10–12^, their origin and their underlying clinical significance remains elusive. The assumption is often that these antibodies originate from incidental, unknown exposures to animal antigen and that they wax and wane over time^13,14^, but most accounts are anecdotic as they come from individual case reports. Moreover, their serum duration is unknown, and such information can provide clues to their origin, and guide the diagnostic and post-diagnostic management of patients with these antibodies.

This study combined a prospective and a retrospective design to assess the clinical significance and interconnection of heterophilic antibodies, anti-Fab and anti-F(ab′)_2_-autoantibodies in dogs. In the prospective part, we followed up a cohort of dogs with heterophilic antibodies against mouse IgG over 2 years. A questionnaire was designed and used to inquire dog owners about any changes in their dogs’ health status during the last 2 years, as well as any known exposures to mice. Follow-up sampling was performed to determine the duration of these antibodies in serum. In the retrospective part, we tested serum samples for the presence of anti-Fab and anti-F(ab′)_2_-autoantibodies and related autoantibody levels with the presence of heterophilic antibodies. Moreover, we related anti-Fab and anti-F(ab′)_2_-autoantibody levels with age, sex, breed and health status. The overarching aims of this study were to elucidate the relation between anti-Fab/F(ab′)_2_-autoantibodies and heterophilic antibodies in dogs and to investigate their possible origin and clinical significance.

## Results

### Survey response

Out of 107 dogs sampled in 2017, 73 owners of a total of 95 dogs were contacted via telephone or e-mail. Nine owners of two dogs, two owners of three dogs, one owner of five dogs and one owner of six dogs were contacted. For seven euthanized dogs, responses were inferred from their medical records. The overall response rate was 73% (75/102), including all completed questionnaires via telephone or e-mail, and inferred responses from medical records. Responses were obtained from seven owners of two dogs, two owners of three dogs and one owner of five dogs. Non-responses (27/95) included owners that were contacted but did not respond to e-mails or telephone calls. Five owners were unreachable due to lack of contact information, and these were not factored into the response rate.

### Breed, sex and age

In total, 75 dogs participated in the questionnaire study. There were 44 Bernese mountain dogs (58.7%) and 33 Labrador retrievers (41.3%). There were 29 intact females, 14 neutered females, 19 intact males, and 13 neutered males. Nine of the dogs (8%) had been neutered since 2017. The median age for all participating dogs was 5 years. The median age was 6 years for dogs testing positive for heterophilic antibodies and 5 years for negative dogs.

### Clinical disease

**Figure 1:**
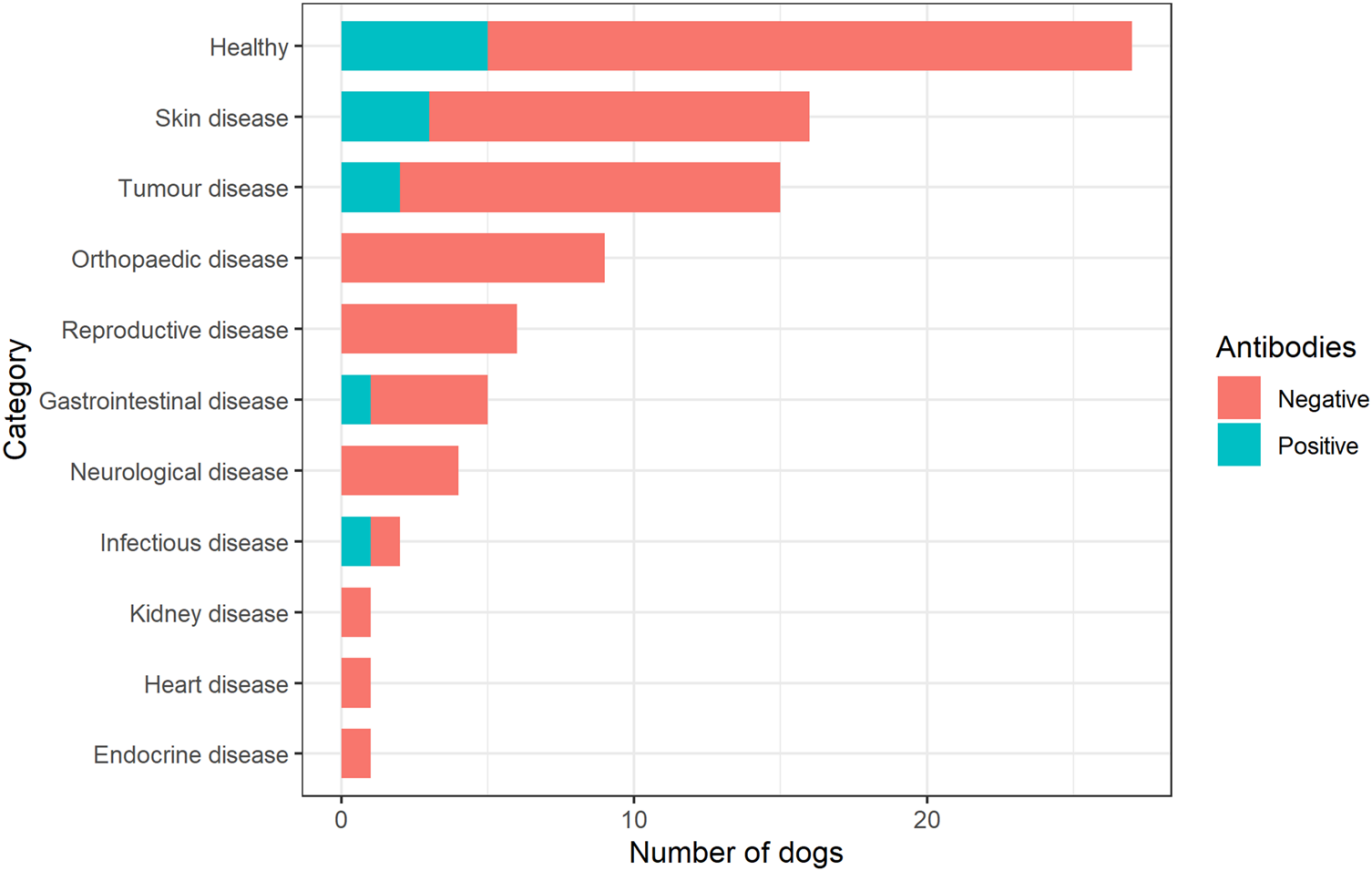
Allocation of diagnoses for different disease categories, including healthy dogs. Stacked bars are separated into two groups based on dogs testing positive or negative for heterophilic antibodies in 2017.

Within 2 years, 61.3% of the dogs had been investigated for disease, out of which 23.9% were included in multiple disease categories. The proportions of positive and negative dogs within the different disease categories are displayed in Fig. 1.

The most common disease category for Bernese mountain dogs were neoplastic disease (22.7%) and for Labrador retrievers orthopaedic disease (25.8%). In total, 13.3% of dogs had received immunosuppressant medication, 12.3% of dogs without heterophilic antibodies 12.3% and 20% of dogs with heterophilic antibodies.

### Mortality

The mortality within 2 years for the disease categories was 60% for neoplastic disease (N = 15), 6% for skin disease (N = 16), 10% for orthopaedic disease (N = 10), 22% for reproductive disease (N = 9), 20% for gastrointestinal disease (N = 5), 25% for nervous disease (N = 4), 50% for infectious disease (N = 2) and 100% for kidney disease (N = 1). For endocrine disease (N = 1) and cardiac disease (N = 1), the mortality was 0%. There was no difference between breeds within the disease categories. Seventeen dogs (22.7%) had been euthanized since the 2017 study, 11 Bernese mountain dogs (25%, median age 8 years) and 6 Labrador retrievers (19%, median age 9 years).

### Dogs with heterophilic antibodies

Ten out of 12 dogs that tested positive in 2017 participated in the questionnaire, and follow-up samples were obtained from seven of these dogs. Six out of seven dogs positive in 2017 remained positive in 2019, while three out of 15 dogs negative in 2017 tested positive in 2019. The age, breed, sex, anamnestic information and antibody reactivities for these dogs are presented in Table 1.

**Table 1:** Breed, age, sex and clinical signs/diagnosis for dogs testing positive for heterophilic antibodies either in 2017 or 2019. Reactivity to mouse IgG in 2017 and 2019 is given.

Dog ID	Age (year)	Sex	Clinical signs/diagnosis		Antibody reactivity	
			2017	2019	2017	2019
B1	9	MN	Protein-losing enteropathy	Liver adenoma or carcinoma^a^	Fc	N/A
B5	8	M	Mastocytoma	Mastocytoma	Fc	Fc
B9	12	FN	Anterior cruciate ligament injury	–	Whole IgG	Whole IgG, Fc
B10	7	FN	Lipoma	Granulocytic anaplasmosis, Lyme borreliosis	Whole IgG, F(ab′)_2_	Whole IgG
B11	5	FN	–	–	Whole IgG	–
B19	3	F	–	Superficial pyoderma	Whole IgG, F(ab′)_2_	Whole IgG, F(ab′)_2_
B24	4	F	–	Superficial pyoderma	–	F(ab′)_2_
B25	6	M	–	Oral blisters, pigmentation loss and widespread redness	Whole IgG	Whole IgG, F(ab′)_2_
B28	9	M	–	Gingival neoplasm	–	Fc
B42	5	MN	–	–	Whole IgG, F(ab′)_2_	Whole IgG, F(ab′)_2_
B44	3	F	–	Abnormal heat cycle, lameness	–	Fc
B51	3	F	–	–	Whole IgG, F(ab′)_2_	N/A
L9	5	MN	Polyuria/polydipsia	–	Whole IgG	N/A

*Bx* Bernese mountain dog, *Lx* Labrador retriever, *F* female, *M* male, *N* neutered, *N/A* dog not sampled.

^a^The dog was euthanized during the follow-up period.

### Exposure to mice

Owners of nine out of 10 dogs that tested positive for heterophilic antibodies against mouse IgG in 2017 reported no observed contact with mice. Information on mouse contact was unavailable for the tenth dog, which had been euthanized during the follow-up period, and the response corresponding to this dog was inferred from available medical records. None of the owners of the three dogs positive for heterophilic antibodies only in 2019 reported observed contact with mice. Out of the dogs testing negative for anti-mouse antibodies in 2017, 10.7% (7/65) of the owners reported observed contact with mice.

### Relationship between disease and heterophilic antibodies

There was no significant relationship between the presence of heterophilic antibodies and clinical diagnosis or signs of disease (*P* = 0.5), nor was there any significant relationship between heterophilic antibodies and any of the specific disease cohorts.

### Anti-Fab and anti-F(ab′)_2_-autoantibodies

Fifty-seven samples were assayed for IgG anti-Fab and anti-F(ab′)_2_-autoantibodies. Thirty samples were from Bernese mountain dogs, 27 from Labrador retrievers, in total 48 dogs (nine dogs contributed two samples). The two samples were collected 2 years apart. All samples were collected in 2017 or 2019. The median age of tested dogs was 4 years (7 months–12 years). There were 18 intact males and six neutered males, 18 intact females, and six neutered females. Thirty-three samples were from healthy dogs and 24 samples from dogs with clinical diagnosis or signs of disease.

There was no significant relationship between levels of IgG anti-Fab-autoantibodies and breed, sex, cancer diagnosis or presence of heterophilic antibodies. Levels of IgG anti-Fab-autoantibodies were increased in dogs with a clinical diagnosis or clinical signs of disease (*P* = 0.03). There was no significant relationship between levels of IgG anti-F(ab′)_2_-autoantibodies and breed, sex, general health status, cancer diagnosis or presence of heterophilic antibodies.

**Figure 2:**
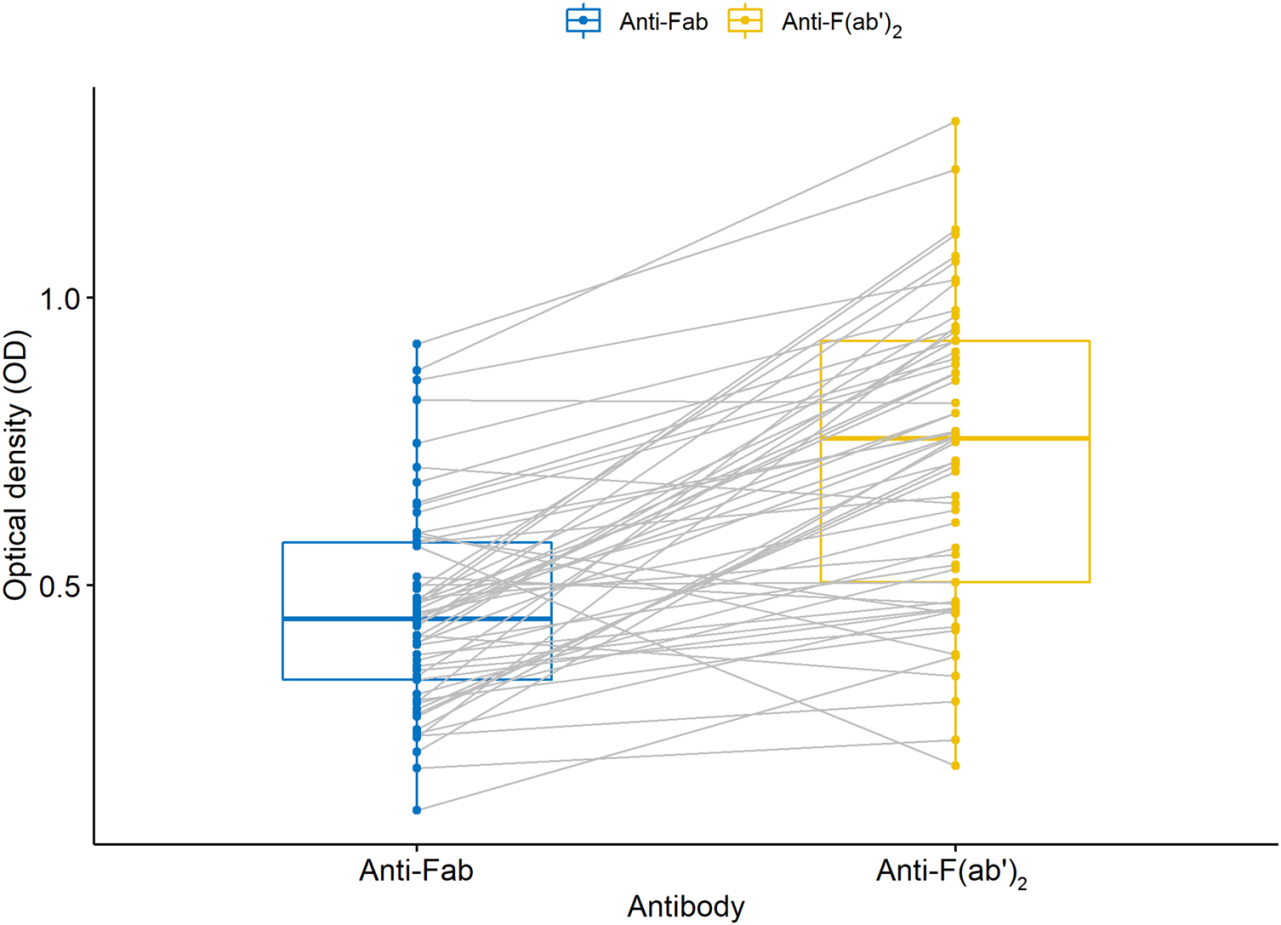
OD values for IgG anti-Fab and anti-F(ab′)_2_-autoantibodies in 57 dog samples. IgG anti-F(ab′)_2_-autoantibody levels were significantly higher than anti-Fab-autoantibody levels (*P* < 0.01).

**Figure 3:**
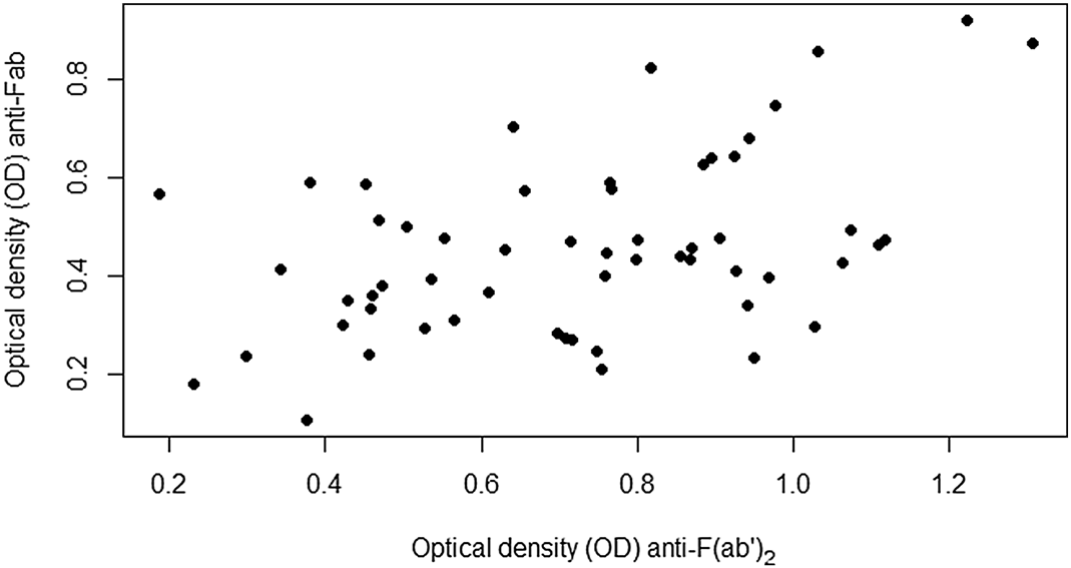
OD levels of IgG anti-Fab and anti-F(ab′)_2_-autoantibodies correlated with Spearman’s rank correlation coefficient. There was a low positive correlation in OD (r_s_ = 0.39).

Levels of anti-F(ab′)_2_-autoantibodies were higher (*P* < 0.01) than levels of anti-Fab-autoantibodies (Fig. 2). There correlation between levels of anti-Fab and anti-F(ab′)_2_-autoantibodies was r_s_ = 0.39 (*P* < 0.01; Fig. 3). The correlation between age and anti-Fab-antibodies was r_s_ = 0.13 (*P* = 0.34) and anti-F(ab′)_2_-autoantibodies r_s_ = − 0.18 (*P* = 0.19).

**Figure 4:**
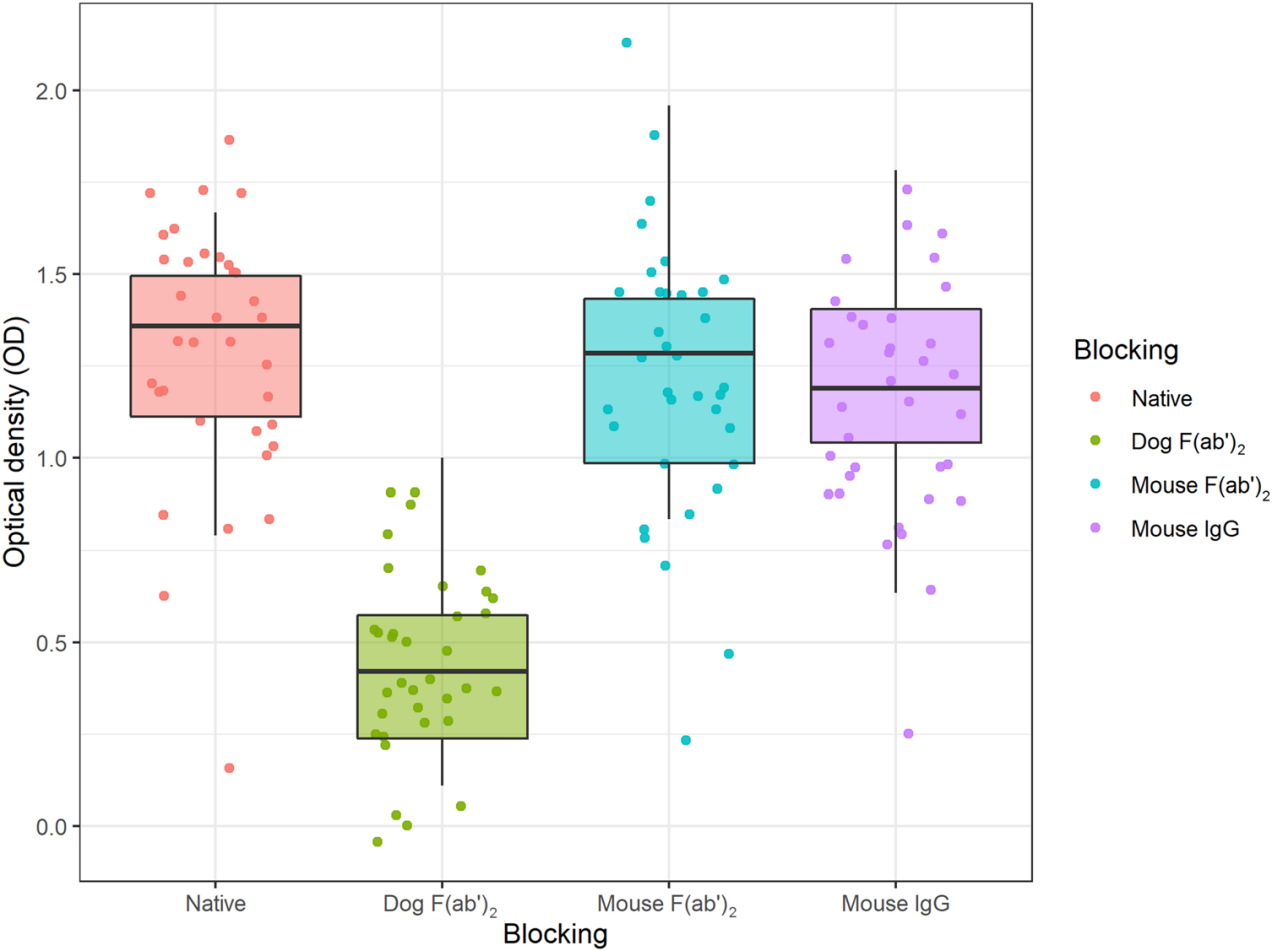
Thirty-five samples were assayed for IgG anti-F(ab′)_2_-autoantibodies native and pre-blocked with 0.5 mg/mL dog F(ab′)_2_, mouse F(ab′)_2_ and mouse IgG. There was a significant overall effect in replicates blocked with dog F(ab′)_2_, but not in replicates blocked with mouse IgG nor mouse F(ab′)_2_.

Thirty-five samples analyzed for anti-F(ab′)_2_-autoantibodies were blocked with 0.5 mg/mL dog F(ab′)_2_, mouse IgG and mouse F(ab′)_2_ (Fig. 4). The treatment with dog F(ab′)_2_ had an overall inhibitory effect on signal (*P* < 0.01), but treatment with mouse IgG (*P* = 0.14) and mouse F(ab′)_2_ (*P* = 0.41) did not have an overall effect on the signal. At a prediction interval of 99.99%, dog F(ab′)_2_ had an inhibitory effect on 35/35 samples (100%). Anti-F(ab′)_2_-autoantibodies cross-reacted with mouse IgG in 3/35 samples (9%) and mouse F(ab′)_2_ in 3/35 samples (9%). Three out of 26 samples (12%) without previously detected heterophilic antibodies cross-reacted with mouse IgG or mouse F(ab′)_2_.

There was no cross-reactivity to mouse IgG nor mouse F(ab′)_2_ in 6/9 samples (67%) with previously detected heterophilic antibodies. Out of these six samples, three were from healthy dogs. Three samples were from dogs with clinical disease or signs of disease; one dog was diagnosed with Lyme borreliosis and anaplasmosis, one with mastocytoma and one dog had allergy-like clinical signs (oral blisters and pigmentation loss).

In 3/9 samples (33%) with previously detected heterophilic antibodies, autoantibodies with cross-reactivity to mouse IgG or mouse F(ab′)_2_ were found. Out of these three samples, two were from dogs diagnosed with a disease (one with superficial pyoderma and one with mastocytoma) and one was from a healthy dog.

## Discussion

The overarching aims of this study were to examine the relation between anti-Fab/F(ab′)_2_-autoantibodies and heterophilic antibodies in dogs and to investigate their possible origin and clinical significance. We performed an anamnestic follow-up of previously studied dogs after 2 years, using a questionnaire distributed to owners of dogs with and without heterophilic antibodies, as well as follow-up sampling and detection of anti-Fab and anti-F(ab′)_2_-autoantibodies in these samples using an in-house ELISA.

To our knowledge, this is the first description of canine anti-Fab and anti-F(ab′)_2_-autoantibodies. Our results indicate that they are widespread in the dog population, at least within the two tested breeds. Anti-F(ab′)_2_-autoantibodies are nearly universal in the human population^15^. Prevalence figures of 98% in study populations consisting of healthy individuals and individuals diagnosed with a disease have been reported^16^. The finding that F(ab′)_2_-autoreactivity is generally more abundant than Fab-autoreactivity is also consistent with previous observations in people^17,18^. Proposed roles for anti-F(ab′)_2_-autoantibodies include clearance of proteolyzed antibodies^19^^,^ mitigation of B cell responses in autoimmune disease^20^ and restoration of Fc-mediated effector functions in proteolytically inactivated IgG^21^. Serum titers are often low^22^^,^ possibly reflecting a low-level naturally occurring autoimmunity. Several lines of evidence suggest that most of the reactivity in canine anti-Fab and anti-F(ab′)_2_-autoantibodies targets cryptic epitopes in the IgG hinge region. First, the reactivity to F(ab′)_2_-fragments transcended the reactivity to Fab-fragments significantly. The only structural difference between a Fab- and a F(ab′)_2_-fragment is that F(ab′)_2_-fragments retain the lower part of the IgG hinge. A significant portion of the binding of the anti-F(ab′)_2_-autoantibodies is therefore derivable to the lower hinge region of IgG, which is a location for cryptic epitopes revealed by several proteolytic enzymes. Second, there was only a low positive correlation between the levels of anti-Fab and anti-F(ab′)_2_-autoantibodies, suggesting that they predominantly target mutually exclusive epitopes. Thus it seems likely that anti-Fab-autoantibodies also bind to cryptic epitopes in the upper hinge region, as these would not be present on F(ab′)_2_-fragments. Finally, because the total serum levels of IgG are significantly higher than the levels of anti-Fab and anti-F(ab′)_2_-autoantibodies of the IgG isotype, we would not expect to be able to detect these antibodies if they formed immune complexes with circulatory IgG in vivo, as they would then be cleared from circulation by the mononuclear phagocyte system. If the autoantibodies target epitopes on Fab- and F(ab′)_2_-fragments that are hidden on the intact IgG molecule, this could explain how they can avoid being depleted from circulation in vivo.

To date, all approved therapeutic mAbs are of the IgG isotype. Full-length IgG binds to antigen with its Fab region and interacts with humoral and cellular components of the immune system with its Fc region. The Fc-mediated effector functions, including antibody-dependent cellular cytotoxicity (ADCC), are toxic to targeted cells. While beneficial in particular treatments, these functions may also cause adverse side effects in other mAb treatments, in which case a therapeutic antibody fragment can be generated by removing the Fc region. Antibody fragments can be advantageous in treatments that benefit from a shortened plasma half-life and improved penetration into solid tumours^23,24^. They are likely to receive consideration as therapeutic agents in the development of immune checkpoint inhibitors, since some checkpoint therapies, including programmed cell death 1 (PD-1) inhibitors, are incompatible with Fc-mediated effector functions^25^. However, pre-existing anti-Fab and anti-F(ab′)_2_-autoantibodies jeopardize the safety and efficacy of therapeutic antibody fragments. If anti-Fab or anti-F(ab′)_2_-autoantibodies are present in the patient upon drug administration, they might react with the therapeutic antibody fragments and restore their ADCC function by providing their own Fc region as a surrogate for immune cells expressing FcγRIIIA-receptors^21,26^. There is documentation of systemic side effects due to anti-Fab/F(ab′)_2_-autoantibodies in preclinical and clinical trials. In a preclinical study, anti-F(ab′)_2_-autoantibodies caused severe thrombocytopenia in 5 of 18 cynomolgus monkeys by reacting with a F(ab′)_2_ therapeutic mAb^27^^,^ and a clinical trial was terminated due to cytokine release initiated by autoantibodies against the Fab region of an anti-TNFR1 antibody^28^. The widespread occurrence of anti-Fab and anti-F(ab′)_2_-autoantibodies in dogs present an obstacle to the use of Fab- and F(ab′)_2_-fragments in canine immunotherapies and other therapeutic agents may be required to block molecular targets while avoiding the triggering of Fc effector functions.

In drug development, immunogenicity is assessed by the detection of anti-drug antibodies (ADAs) using immunogenicity assays. Anti-F(ab′)_2_-autoantibodies are a risk factor for interference in these assays. An immunogenicity test is often configured as an immunometric assay with the therapeutic antibody as capture and detection antibody, detecting ADAs that form in response to antibody administration. Because rheumatoid factors are common in both people and dogs, interference through binding to the Fc region of the therapeutic antibody is common in these assays, and it is therefore sometimes recommended to use F(ab′)_2_-fragments of the therapeutic antibody to capture and detect ADAs^5^. However, this may introduce anti-F(ab′)_2_-autoantibodies targeting cryptic epitopes in the hinge region as a new source of interference. In this scenario, a therapeutic mAb with low actual immunogenicity may falsely appear to be highly immunogenic, since pre-existing anti-F(ab′)_2_-autoantibodies against cryptic epitopes are not ADAs, and do not bind to intact full-length IgG. Addition of irrelevant dog F(ab′)_2_ could limit or resolve the problem with interference from anti-hinge-autoantibodies in immunogenicity assays. However, high concentrations may be needed since 0.5 mg/mL dog F(ab′)_2_ had significant but limited effect on blocked samples. It might also be necessary to heat-treat the F(ab′)_2_-fragments to increase the blocking efficiency^29^.

There is some indication that the relationship between pre-existing anti-Fab/F(ab′)_2_-autoantibodies and disease runs deeper than the risk for immunogenicity in disease treatment with therapeutic antibodies. The presence of pre-existing anti-IgG autoantibodies may itself be associated with the diseases that these therapeutic antibodies are intended to treat. According to a recent theory, the human immune system produces anti-F(ab′)_2_-autoantibodies to counteract attempted immune escape when cancer-associated proteases inactivate IgG through cleavage in the hinge region^6^. However, there was no connection between anti-Fab or anti-F(ab′)_2_-autoantibody levels and cancer diagnosis, nor was there any significant relationship with sex, breed or age. This observation, in combination with their widespread occurrence, favours the hypothesis that several different pathways can induce them. Anti-Fab-autoantibody levels were increased in dogs with a diagnosed disease or clinical signs of disease. Increased serum titers of anti-Fab-autoantibodies have, for example, been reported in patients with HIV disease^30^^,^ squamous cell carcinomas and adenoid cystic carcinomas of the head and neck^31^^,^ and gram-positive sepsis^15^^,^ motivating future investigations directed at disease-associated enzymes capable of cleaving canine IgG in the upper hinge region in vivo. Although rheumatoid factors are associated with autoimmune disease in dogs, clinical indications for testing of canine rheumatoid factors are rare due to test insensitivity^32^. Since anti-Fab-autoantibodies and rheumatoid factors both target the IgG molecule, it is conceivable that anti-Fab-autoantibodies could represent a diagnostic complement or alternative to rheumatoid factors in testing for canine autoimmune disease.

Anti-IgG autoantibodies can present as heterophilic antibodies in immunoassay analysis by cross-reacting with allospecific IgG, often mouse IgG. It is, therefore, of value to elucidate a potential connection between autoantibodies and heterophilic antibodies in dogs. Because autoantibodies may be disease-associated and autoantibodies and heterophilic antibodies can be related, by logical extension, there might also be a connection between heterophilic antibodies and clinical disease. Autoimmune disease is a potential link due to the connection between heterophilic antibodies and autoantibodies, but other studies have demonstrated connections between, for example, *E. coli* infection and interference from broadly reactive antibodies against bacterial surface epitopes^33^. Similarly, immunogenic cancer forms such as melanoma and malignant lymphoma could potentially induce antibodies against mutated surface proteins on cancer cells. An overrepresentation of heterophilic antibodies has previously been found in human patients diagnosed with cancer^34^. In the prospective follow-up, we found that some owners of dogs positive for heterophilic antibodies reported clinical signs or diagnoses that are consistent with immune-mediated diseases, including a Labrador retriever with diagnosed hypoadrenocorticism and a Bernese mountain dog presenting with allergy-like clinical signs. Ultimately though, this study could not prove a correlation between clinical diagnosis or clinical signs of disease and the presence of heterophilic antibodies. The cross-reactivity between canine F(ab′)_2_ and mouse IgG or mouse F(ab′)_2_ was sporadic (six out of 35 analyzed samples), and anti-Fab or anti-F(ab′)_2_-autoantibody levels were not increased in dogs with heterophilic antibodies. In the majority of samples with heterophilic antibodies that were evaluated for cross-reactivity to canine F(ab′)_2_ (five out of nine), the presence of heterophilic antibodies against mouse IgG could not be reconciled with any proposed risk factor, including observed contact with mice, diagnosed cancer or autoimmune disease, nor with autoreactivity to IgG. The origin of heterophilic antibodies in dogs thus remains mostly unknown, although a minority of samples evaluated for autoreactivity cross-reacted with canine F(ab′)_2_, establishing canine anti-F(ab′)_2_-autoantibodies as a potential source of immunoassay interference. However, it should be kept in mind that anti-F(ab′)_2_-autoantibodies appear to be nearly ubiquitous in dogs, and it is not known what distinguishes cross-reactive canine anti-F(ab′)_2_-autoantibodies from autoantibodies that do not cross-react with mouse IgG. We found that heterophilic antibodies with initial reactivity to mouse IgG were detectable after 2 years in 85% of the dogs (six out of seven). The persistence of canine heterophilic antibodies in serum is a risk factor for repeated immunoassay interference in dog patients with these antibodies. In a clinical setting, suspected cases of immunoassay interference should be noted in the patient’s medical records, and extra caution is advised in the interpretation of any subsequent immunoassay test results from patients with heterophilic antibodies.

The reaction pattern of the heterophilic antibodies was, in essence, identical to the initial testing, i.e. no F(ab′)_2_-reactive antibodies had shifted or expanded to become Fc-reactive antibodies, or vice versa. The long serum duration and the binding properties of the antibodies are inconsistent with the idea that they originate from exposure to mice, which is a possible source of heterophilic antibodies against mouse IgG^35^. Prolonged antibody responses are mediated by long-lived plasma cells (LLPCs) or memory B cells^36^. Memory B cells need to be reactivated by repeated exposure to antigen to secrete antibodies into the circulatory system. The serum half-life for canine IgG is unknown, but the canine IgG subclasses display many structural and functional properties analogous to human IgG^37^^,^ which has a serum half-life of 7–21 days. The presumably narrow window of time in which the exposure to mice would need to occur, combined with the lack of observed contact with mice reported by owners, does not suggest an exposure origin. It is possible that LLPCs continuously produce the antibodies after initial contact with mice before the first sampling, but if that is the case, all of the dog owners must have overlooked the contact. Moreover, the heterophilic antibodies exhibit a prolonged selective binding to either Fc- or F(ab′)_2_-fragments, while immunization to mouse IgG results in reactivity against both of these fragments^8^.

There are some limitations to this study, most notably a small sample size within the positive cohort consisting of dogs with heterophilic antibodies, which limits the statistical power for the prospective evaluation of the clinical significance of heterophilic antibodies. We only included two dog breeds in the study, so the results cannot be fully generalized to the entire dog population. Different disease categorizations could also have led to different results and interpretations. We investigated the possibility that exposure to foreign antigen can induce the formation of heterophilic antibodies by inquiring dog owners about any observed contact with mice. Additional information on the source of the antibodies could potentially have been gained by also asking about environmental factors such as rural versus urban housing, walking routines (strictly leashed vs unleashed), or factors relating to dietary regimens, such as types or brands of food. Immunization via the gut has been speculated to be a source of heterophilic antibodies in humans^38^^,^ but it is unclear if this proposed source can be reconciled with the concept of oral tolerance to food protein^39^. For the time being, the origin and clinical significance of heterophilic antibodies in dogs remains elusive. Nevertheless, the study provides some basic knowledge that can be useful for the interpretation of immunoassay analysis as well as in the development of future therapeutic antibodies. If history is any indication, dogs are very likely to become the primary recipients of novel cancer treatments in veterinary medicine. The FDA has currently approved two mAbs for the treatment of cancer in dogs, and several others are in the clinical pipeline^2^. Unlike many other immunotherapies, therapeutic mAbs do not require personalization for each patient, and mAb production techniques are well-established, making mAb treatment one of the currently most feasible forms of veterinary immunotherapy. Despite these reasons for optimism, the promise of therapeutic veterinary mAbs remains as yet unfilled, and extensive research will be needed to certify the safety and efficacy of prospective veterinary mAb treatments.

## Conclusions

Anti-Fab and anti-F(ab′)_2_-autoantibodies reactive with cryptic epitopes in the hinge region of IgG were widespread in the studied dogs. Their presence needs to be considered in the development of therapeutic antibodies as they may affect drug safety and efficacy, as well as clinical immunogenicity testing. Serum presence of canine heterophilic antibodies to mouse IgG is not uncommonly stable for 2 years. No owners of dogs with heterophilic antibodies against mouse IgG reported observed contact with mice, suggesting a non-contact aetiology in these antibodies. Some instances of canine heterophilic antibodies can be explained by autoreactivity, as some anti-F(ab′)_2_-autoantibodies cross-reacted with mouse IgG. However, the origin of canine heterophilic antibodies remains generally elusive, as most instances of their presence could not be reconciled with any purported risk factor for heterophilic antibodies, including cancer, autoimmune disease, nor autoantibodies to IgG.

## Methods

### Approval of study

Informed written consent was obtained from all private dog owners. The study was approved by the Uppsala Animal Ethical Committee (C136/13), and all experiments were performed in accordance with relevant guidelines and regulations. Because no sensitive personal data was collected, the questionnaire is not reviewable by the Swedish Ethical Review Authority according to Swedish law (2003:460) and there was thus no specific ethical approval obtained for experimentation on humans.

### Questionnaire

The questionnaire was distributed to owners of Bernese mountain dogs and Labrador retrievers previously participating in a study in 2017. Bernese mountain dogs were selected for their high frequency of heterophilic antibodies, while Labrador retrievers have a lower frequency of heterophilic antibodies. Dog owners with a supplied e-mail address received an invitation via e-mail to participate in the follow-up study. The questionnaire was hosted by a commercial online survey platform and open for participation for 6 weeks in October–November 2019. Owners were asked if their dogs had received veterinary care for disease or suspected disease after the year 2017, and if that was the case, for which type(s) of disease within the categories skin disease, kidney disease, gastrointestinal disease, nervous disease, tumour disease, orthopaedic disease, cardiac disease, and other disease. They were given the option to state which specific disease(s) or disease signs the dogs were investigated for, and whether they had received a diagnosis, in a free-text box. Next, owners were asked which year the investigation occurred. Owners with dogs not investigated for disease skipped ahead to answering if the dogs had received any pharmacological treatment after the year 2017. For owners without a dog investigated at a veterinary clinic, it was possible to state any potential self-diagnosis. Owners were asked to state whether their dog was still alive or not. If the dog was no longer alive, owners were asked for the time and cause of death, if the cause was known. Owners were asked whether their dogs have been observed chasing or otherwise having contact with mice. Finally, owners were given the option to volunteer their dogs for follow-up blood sampling. Telephone interviews were conducted with owners that had not provided an e-mail address, using the same questions.

### Serum samples

Samples were obtained by venipuncture of the cephalic vein of participating dogs. The samples were centrifuged for 10 min at 3,000 rpm and frozen at − 20 °C before analysis. None of the samples had clear visible signs of hemolysis, lipemia or bilirubinemia.

Fifty-seven serum samples collected in 2017 and 2019 were used in the screening for anti-Fab and anti-F(ab′)_2_-autoantibodies. Thirty samples were collected from 24 Bernese mountain dogs, 6 with two samples collected in 2017 and 2019, 6 with a single sample collected in 2017 and 12 with a sample collected in 2019. Twenty-seven samples were from 24 Labrador retrievers, 2 with two samples collected in 2017 and 2019, 22 with a single sample collected in 2017 and 1 with a sample collected in 2019.

In total, 22 dogs were sampled in 2019, of which seven were positive for heterophilic antibodies in 2017, and 15 were negative for heterophilic antibodies. All of these follow-up samples were tested for heterophilic antibodies.

Thirty-five samples from the 2017 and 2019 samplings were used in the blocking experiment. Of these, 13 samples were from 13 Labrador retrievers, and 22 samples were from 17 Bernese mountain dogs. Nine of the samples had previously tested positive for heterophilic antibodies. The samples were selected to represent a variety of health statuses, ages and sexes in all three experiments.

### Assays for detection of IgG anti-Fab and anti-F(ab′)_2_-autoantibodies.

Microtiter plates were coated with 2 µg/mL of canine Fab-fragments (MyBioSource, MBS537495) and F(ab′)_2_-fragments (MyBioSource, MBS537274) diluted in NaHCO_3_ (0.05 M, pH 9.6). The plates were washed four times with Tween-20 diluted in PBS (200 μL, 0.05%). Neat serum samples were incubated as duplicates for 90 min at RT. Four wells on each plate were incubated with PBS as a negative control. Autoantibodies were detected with biotinylated Fc-specific goat anti-canine IgG (Sigma Aldrich), diluted 1:20,000 in PBS. The wells were then incubated with 1:5,000 streptavidin-HRP for 90 min, washed and incubated with TMB for approximately 8 min in the dark before the reaction was stopped with H_2_SO_4_ (100 µL). Plates were read at 450 nm in a photometric microplate reader. All incubations were made with volumes of 50 μL except for the TMB, of which 100 μL was added.

The ELISA procedure for the blocking experiment was performed as above, with a 1:10,000 dilution of the anti-canine IgG detection antibody. Samples were blocked with 0.5 mg/mL of canine F(ab′)_2_, mouse IgG and mouse F(ab′)_2_, amounting to a 1:2 dilution factor. Native replicates were diluted 1:2 with PBS. A 99.99% prediction interval for the difference in OD after blocking was established by calculating the intra-assay CV based on four standard deviations (CV_99.99%_).

### Detection of heterophilic antibodies against mouse IgG.

Heterophilic antibodies against mouse IgG were detected using a previously described method^8^. Briefly, microtiter plates were coated with 2 μg/mL mouse IgG, 50 μL of neat serum samples were added to the wells, and an HRP-conjugated monoclonal mouse IgG1k (MyBioSource, MBS592181) diluted 1:200 was used for detection. Plates were washed and read as described above.

### Statistical analysis

Fisher’s exact test was used to test for correlation between disease cohorts and heterophilic antibody presence. Differences in levels of autoantibodies between sex, breed and disease cohorts were tested using the Wilcoxon rank-sum test. The significance level was α = 0.05.

Spearman’s rank correlation was used to evaluate the association between autoantibody levels and age as well as between levels of anti-Fab and anti-F(ab′)_2_-autoantibodies. The correlation coefficients were interpreted according to a rule of thumb^40^.

## Data Availability

The submitted manuscript includes all data generated in this study.
